# Person-Centred Care: State-of-the-Art and Future Perspectives

**DOI:** 10.1007/s11897-025-00702-3

**Published:** 2025-04-11

**Authors:** Hanna Gyllensten, Matilda Cederberg, Sara Alsén, Elin Blanck, Lilas Ali, Andreas Fors, Håkan Hedman, Laura Pirhonen Nørmark, Karl Swedberg, Inger Ekman

**Affiliations:** 1 Institute of Health and Care Sciences, Sahlgrenska Academy, University of Gothenburg, Box 457, 405 30 Gothenburg, Sweden; 2 Centre for Person-Centred Care (GPCC), Sahlgrenska Academy, University of Gothenburg, Gothenburg, Sweden; 3 Department of Psychotic Disorders, Region Västra Götaland, Sahlgrenska University Hospital, Gothenburg, Sweden; 4 Faculty of Caring Science, Work Life and Social Welfare, University of Borås, Borås, Sweden; 5 Department of Affective Disorders, Region Västra Götaland, Sahlgrenska University Hospital, Gothenburg, Sweden; 6 Research, Education, Development and Innovation, Region Västra Götaland, Primary Health Care, Gothenburg, Sweden; 7 Swedish Kidney Association, Stockholm, Sweden; 8 Department of Economics, Centre for Health Economics (CHEGU), University of Gothenburg, Gothenburg, Sweden; 9 Section of Health Services Research, University of Kopenhagen, Kopenhagen, Denmark; 10 Department of Clinical and Molecular Medicine, Sahlgrenska Academy, University of Gothenburg, Gothenburg, Sweden; 11 Department of Medicine, Geriatrics and Emergency Medicine, Region Västra Götaland, Sahlgrenska University Hospital/Östra, Gothenburg, Sweden

**Keywords:** Person-centred care, Heart failure, Pulmonary disease, Chronic obstructive pulmonary disease, Acute coronary syndrome, Common mental disorders

## Abstract

**Purpose of Review:**

Many countries prioritise the implementation of person-centred care. This study examines the progression of research in person-centred care, specifically focusing on using complex interventions within intricate contexts. It aims to explore how previous experiences can inform and shape subsequent projects. The review was based on five studies from our research group, encompassing 1099 patients, resulting in 41 peer-reviewed scientific publications. Most studies focused on patients suffering from chronic heart failure, as well as patients with chronic obstructive pulmonary disease. Additionally, interventions for acute coronary syndrome and common mental disorders were also considered. Analyses included the development of a logical model for person-centred care, an overview of partnership operationalisation, and the establishment of evaluation criteria for the trials. The analyses involved creating a coherent model for person-centred care, examining partnership operationalisation, and establishing trial evaluation criteria.

**Recent Findings:**

Sequential trials build upon their predecessors and add new elements. The studies conducted by clinicians in usual care and in-house by research staff were complementary, providing a deeper understanding of the efficacy and effectiveness of person-centred care. Initiating, working, and safeguarding a partnership between patient and staff was possible, whether through in-person or remote communication. Evaluations followed modern research standards and incorporated past study insights for a more thorough approach.

**Summary:**

This study highlights how the cumulative experience from previous research in person-centred care informs the design and analyses of subsequent projects through an iterative learning process, particularly important for complex interventions in various health care contexts.

**Supplementary Information:**

The online version contains supplementary material available at 10.1007/s11897-025-00702-3.

## Introduction

Many countries are endorsing care models centred on the values and preferences of patients [1]. Person-centred care is one such centredness [2]. The distinguishing feature of person-centred care is its emphasis on living a meaningful life, which sets it apart from other models [3]. Although various forms of centredness can be identified throughout history [1], the implications of transitioning to a more person-centred care model have only been explored recently. The University of Gothenburg Centre for Person-Centred Care (GPCC) (www.gpcc.gu.se) was established in February 2010 and formalised as the first centre in Europe to enhance and coordinate interdisciplinary research in person-centred care. The centre includes health professionals and researchers from several disciplines and a "person council" for patients, relatives, and carers.

In the GPCC, one of the research groups, under the leadership of Inger Ekman, comprises researchers and PhD students from diverse disciplines such as care sciences, pedagogics, psychology, medicine, health economics, and patient representatives. The group has undertaken numerous controlled trials during GPCC's existence as a research centre, encompassing projects devised during the initial and subsequent phases of GPCC studies [4]. These trials use the GPCC model, also referred to as the Gothenburg model [5, 6], for person-centred care. Most studies involved patients with chronic heart failure (CHF) or chronic obstructive pulmonary disease (COPD) but interventions in patients with acute coronary syndrome and common mental disorders were also considered.

This study examines the progression of person-centred care research through sequential trials, where previous experiences inform the development of new projects. Thus, the review intends to provide an understanding of the current state-of-the-art research into person-centred care and inform future research.

## Methods

The review is based on five previous studies by the same research team (Table 1). In total, 1099 patients were enrolled in the five studies, leading to 41 peer-reviewed scientific articles (5 to 12 publications per study). The online supplementary material (sCase 1–5) reports additional information about each study.

**Table 1:** Overview of the studies included in this study

Study	Design	Patient population		Recruitment	Publications
		Inclusion	Exclusion		
I	Controlled before and after design	-Patients diagnosed with **chronic heart failure** (CHF) (ICD 500–509) with worsening symptoms or cardiomyopathy (ICD 420)	-Patients with acute myocardial infarction or chest pain -Age < 50 years, primary valvular disorder, severe concomitant illness (e.g., cancer) -Survival expectancy < 3 months -Planned surgical intervention -Cognitive impairment or reluctance to participate	During hospitali sation	8
II	Randomised controlled trial	-Patients diagnosed with **acute coronary syndrome** (ACS) (ICD = I200, I209 or I21) within 72 h after hospital admission	-Patients aged ≥ 75 years -Currently listed at a private primary care centre or a primary care centre in another region -No permanent address -Planned heart surgery such as coronary artery bypass grafting -Cognitive impairment -Alcohol or drug abuse -Survival expectancy < 1 year -Participating in a conflicting study	During hospitali sation	12
III	Randomised controlled trial	- Patients hospitalised due to worsening **chronic heart failure** (CHF) **and/or chronic obstructive pulmonary disease** (COPD) -Patients > 50 years -Access to a telephone with an active subscription	-Patients with no registered address in the Västra Götaland region -Severe hearing impairment -Cognitive impairment -Current alcohol or drug abuse -Survival expectancy < 1 year -Participating in a conflicting study	During hospitali sation	9
IV	Randomised controlled trial	- Patients with a diagnosis of **chronic heart failure** (CHF) (I50.0-I50.9) **and/or chronic obstructive pulmonary disease** (COPD) (J43.0, J44.0-J44.9) -Listed at one of the participating primary health care centres -Understanding written and spoken Swedish -Having access to a device with an internet connection	-Patients with severe impairment (cognitive or other) preventing the individual from using eHealth support -No registered address (follow-up questionnaires sent by regular mail) -Expected survival < 1 year -Ongoing documented diagnosis of alcohol or drug abuse -Diseases that could interfere with follow-up -Participation in a conflicting study	From primary care units	5
V	Randomised controlled trial	-Currently on sick leave ≤ 30 days for **common mental disorders** (CMD), including mild to moderate depression (F32 and F33), mild to moderate anxiety disorder (F41), reaction to severe stress and adjustment disorders (F43, except posttraumatic stress disorder) -Patients aged 18–65 years -Employed or studying at least part-time during the past 9 months	-No registered address in Sweden -Lack of proficiency in Swedish -Previous sick leave due to depression, anxiety disorders or stress reactions and disorders > 14 days in the past 3 months -Severe impairments preventing the use of eHealth intervention or if the intervention was assessed as a burden -Ongoing alcohol or drug abuse -Severe disease with an expected survival of < 1 year or that could interfere with follow-up -Participating in a conflicting study	From primary care units	7

### Analyses

A logic model for person-centred care was developed to understand what changed between studies [7, 8]. Thereafter, the operationalisation of partnership and development of the evaluations were described, focusing on changes from study to study.

### Patient Involvement

Patient involvement has been an essential component in all the projects. More specifically, in the design phase, the patient representatives in each study played a crucial role in offering insights on the viability of the interventions and research procedures, including factors like the number of items in the questionnaires and the content of the interview guides. The involvement of the patient representative remains crucial in the ongoing project, as they play a vital role in discussing and contextualizing the findings to ensure their relevance for the affected individuals, specifically the patients. In the capacity of a co-author, the patient representative actively participates in group meetings, contributing to the analysis, interpretation of results, and writing of the manuscript.

## Results

**Fig. 1:**
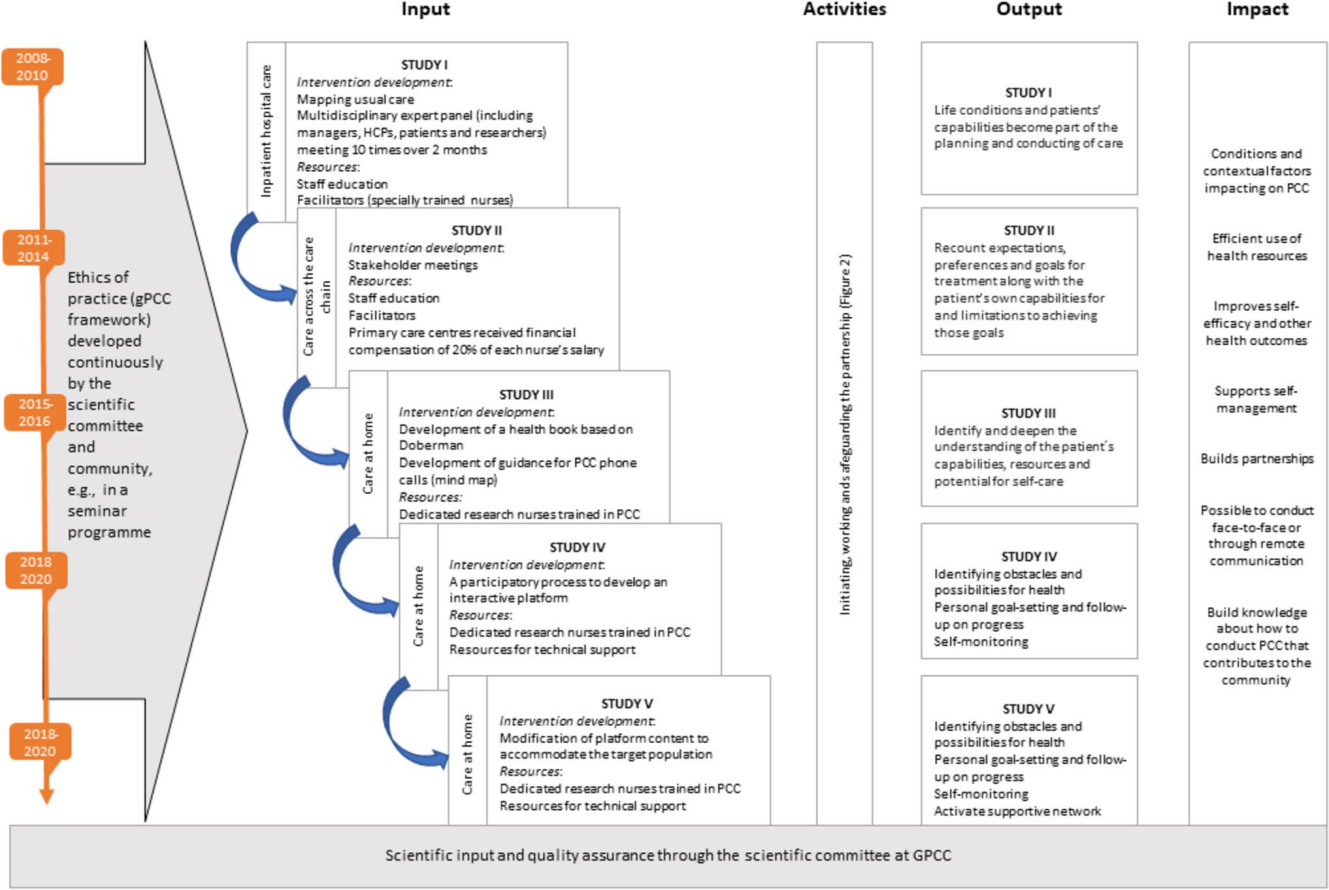
Logic model of person-centred care in the research programme. Abbreviations: gPCC = Gothenburg model for person-centred care; PCC = person-centred care

The logic model (Fig. 1) covers the resources, activities, and effects of each of the five projects and illustrates how each sequential project builds on its predecessor. The patient's usual care provider conducted studies I-II, i.e., clinicians employed in usual care.

Hence, a significant portion of the initial efforts focused on engaging the personnel in the healthcare units to participate in the development of the intervention and the study planning (see input in Fig. 1). The person-centred intervention in study I was developed collaboratively by a team comprising experienced staff nurses, physicians, physiotherapists, occupational therapists, representatives from the local patient association, and the research team. The expert group convened on 10 occasions over 2 months to review usual care practices and discuss and propose measures to align the care with the person-centred care model. All staff (about 300 persons) received a 3-h introduction to the philosophy and theory of person-centred care. Study II implemented a formalised system where dedicated study nurses monitored and supported the staff. They also organised regular meetings to ensure the health care team's active participation and proper intervention delivery. In the hospital-based study (study I), all staff members were actively engaged for at least 2 years, likely facilitating the subsequent implementation process. After the study's conclusion in 2011, the managers implemented person-centred care at the Department of Medicine (6 wards involving approximately 300 employees) at the Sahlgrenska University Hospital (SU). Another study conducted at SU around the same time and with a similar design found that implementing a person-centred pathway for patients with a hip fracture resulted in a 50% reduction in hospital stays. [9]. As a result of these studies, the hospital director at SU (which has 17,000 employees) decided a year later to adopt person-centred care as the hospital's profile.

In studies lll-V the intervention was delivered remotely. The focus was exploring alternative avenues for implementing person-centred care and refining the communicative protocols. As a complement to standard care, the research team conducted these studies, developing and adapting the intervention in close collaboration with the patient's regular care providers. The results of these studies show that increased self-efficacy and a sense of partnership extended beyond face-to-face interactions [10–16]. Accordingly, remote communication can be perceived as an extension of interpersonal connections beyond in-person encounters, mediating the desire for a meaningful existence and fostering a cooperative approach to caregiving. Throughout these studies, the research group, which included patient representatives, consistently convened to discuss the application of person-centred ethics to enhance their practice. This involved actively listening to and evaluating each other's person-centred dialogues with patients.

### Operationalising Partnership

**Fig. 2:**
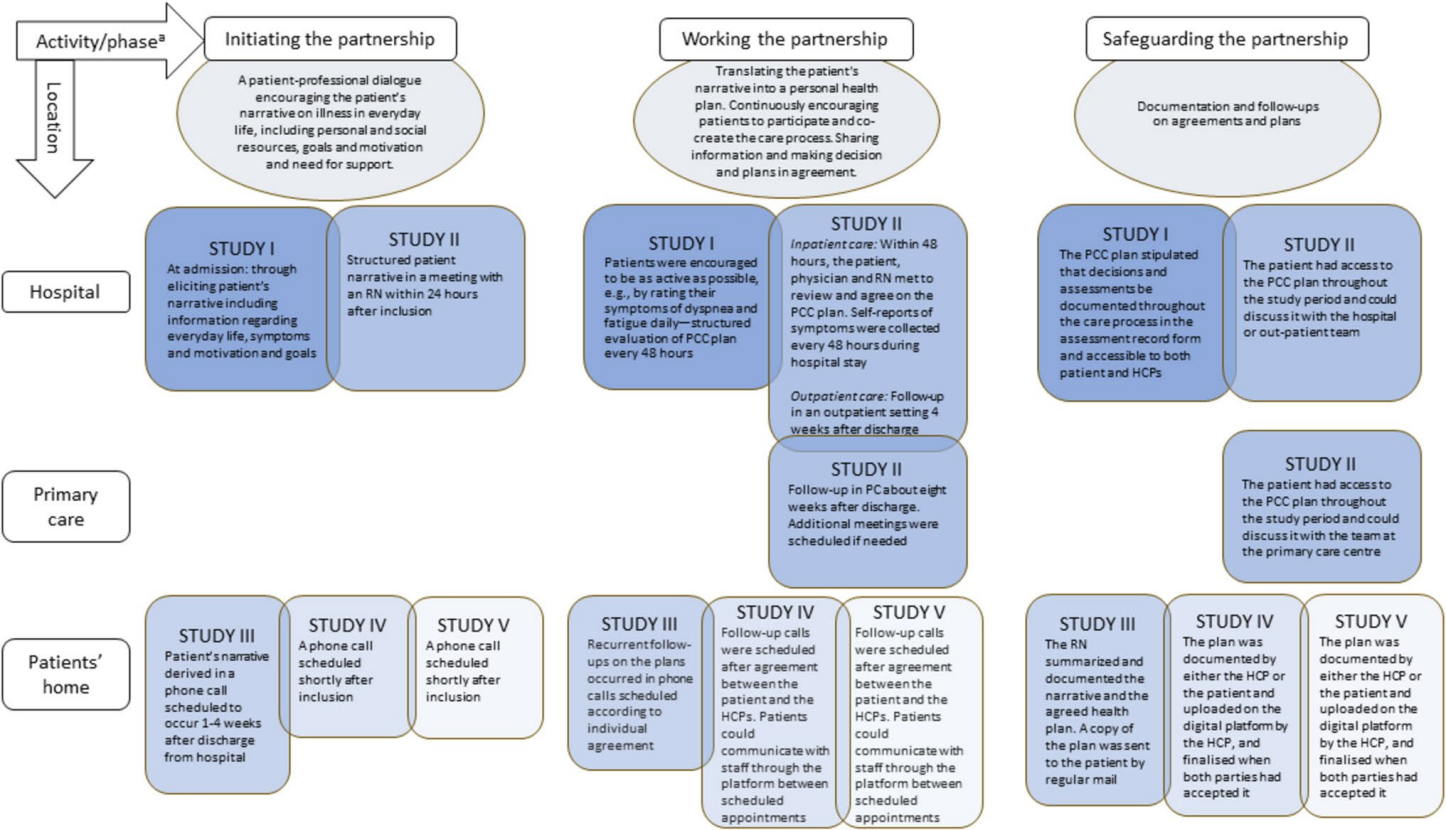
Overview of partnership in the respective interventions. ^a^ The PCC interventions were planned jointly by patient representatives and care professionals in collaboration with the research team. All gPCC professionals had received training in the theory and practice of gPCC through lectures, seminars, and workshops and were given practice in formulating and executing gPCC plans. Abbreviations: gPCC = Gothenburg model for person-centred care; HCP = health care professional; PC = primary care; PCC = person-centred care; RN = Registered nurse

A crucial aspect of these studies, pertained to the operationalisation of the partnership (Fig. 2). The *Gothenburg model* for person-centred care [6] is based on Paul Ricoeur's 'Little ethics,' summarised as "aiming for the good life, *with* and for others in just institutions" [17]. The principles of applied ethics require health care professionals to regard patients as equal partners in the planning and executing of their care [10]. Therefore, person-centred care focused on three cornerstones under the partnership umbrella: i) Initiating the partnership between the patient and health care providers through active listening and open-ended questions, ii) Working the partnership (collaboratively formulating health plans), and iii) Safeguarding the sustainability of the partnership through goal documentation and planning [5]. A common thread among all projects was the emphasis on the partnership between patients and staff*.*

Early on, all projects included one or more occasions for a patient-professional dialogue to *initiate the partnerships*. During these dialogues, patients shared their understanding and personal experiences of their condition and how it impacted their daily life and future outlook. At the same time, healthcare personnel discussed treatment expectations, preferences, and goals with patients, as well as the capabilities and limitations associated with those objectives. The research team would participate in the patient's narrative, primarily by listening and asking follow-up questions and collectively investigating urgent inquiries [18]. Additionally, they attempted to ascertain the patients' desires and capabilities. The patient and staff identified problems (e.g., dilemmas related to taking prescribed medicines, sleeping problems, or returning to work), which were, to some extent, influenced by context and local settings in each project (Fig. 2). A personal health plan was formulated based on this and other clinical information.

*Working the partnership* included routines and infrastructure to facilitate patients being active partners in their health by sharing information and mutually agreeing on decisions. Patients had continuous access to their health plan throughout the study period, allowing them to engage in discussions with the research team involved in each study. The scheduling of meetings and phone calls considered the specific circumstances of each participant without any predefined limitations on the number of interactions. All studies included a component of symptom rating, structured checkups, and revisions of the initial health plan aimed at maintaining and reinforcing (*working*) a partnership between patients and health care providers. In studies I-II, meetings between patients and staff occurred on-site at the hospital. Patients in study II were actively engaged throughout the care process. At each care transition, there was a meeting in which patients and staff followed up and revised the health plan. Studies III-V deliberately excluded face-to-face interactions to examine the possibility of establishing a partnership from a distance. In study III, all patient-staff interactions took place remotely, specifically via phone calls. The intervention also included a health book to promote patient engagement and shared decision-making by encouraging note-taking and personal accountability in achieving their goals. In studies IV-V, interaction was established through phone calls and a web-based platform. The platform allowed for communication in a chat forum between meetings, and patients could manage their illness through symptom tracking, learn more about their condition using links to relevant websites, and inform their supportive network by inviting family or health care professional contacts to their platform.

In all five studies*, safeguarding the partnership* corresponded to activities that ensured that information and agreements were documented in systems (on paper or the web-based platform) and accessible to both patients and staff. The documentation aimed to ensure the agreements and planned activities guided the care process.

### Evaluation of Partnership

Assessment of the studies included a range of outcomes (Table 2) to avoid overlooking any significant changes in the participants' health status. This included diagnosis-specific outcomes such as symptom scales and generic outcomes relevant to the patient populations in each study. Studies II-V incorporated composite endpoints that integrated patient experience and clinical outcomes in line with contemporary practices in cardiovascular trials [19]. Few events were anticipated that required a large patient sample to obtain reasonable power, but using a composite endpoint could limit the sample size needed. While this choice provides a meaningful combination of important clinical and effective outcomes, a notable insight has been that it is also necessary to break down the composite into its constituent elements [20] to understand the study results fully. Moreover, the informants sometimes experienced a burden when completing the questionnaires or mistakenly believed they were part of the person-centred care rather than the evaluation.

**Table 2:** Overview of dimensions examined in the studies

Main category^a^	Sub-category^a^	Measured dimensions	Outcome	Study^b^
Economic	Economic outcomes	**Cost-effectiveness/Cost-utility**^c^	CUA: direct hospital costs	I
			CUA: direct health care and drug costs	III
			CUA: direct health care and drug costs, productivity loss from sick leave	II, IV-V
Clinical	Intermediaries	**Physical functioning**	Activities of daily living (ADL)	I
	Outcomes	**Health care use**	Length-of-stay	I
Humanistic	Intermediaries	**Coping capacity**	Cardiac self-efficacy	II-III
			General self-efficacy	II-V
			Self-Efficacy to Manage Chronic Disease (SEMCD) scale	V
		**Disease activity**	Multidimensional Fatigue Inventory (MFI 20)	II-III, V
			Hospital Anxiety and Depression Scale (HAD)	II-V
			Stress Perceived Stress Scale (PSS-14)	II V
			Shortness of breath (SOB)	III-IV
			CAT (COPD assessment test)	III-IV
			Medical Research Council (MRC) dyspnoea scale	IV
			SMBQ (Shirom-Melamed Burnout Questionnaire)	V
			Self-rated Exhaustion Disorder (s-UMS)	V
		**Physical functioning**	Sheehan Disability Scale	II, V
			Somatic Health Complaints Questionnaire (SHCQ)	II
			International Physical Activity Questionnaire (IPAQ)	
	Outcomes	**Health and well-being**	Kansas City Cardiomyopathy Questionnaire (KCCQ)	I
			EQ-5D (HRQoL)	I-V
		**Return to work**	Level of sick leave	V
		**Satisfaction**	Picker Patient Experience questionnaire (PPE-15 and PPE-3)	II
Other	Treatment modifiers	**Knowledge**	Uncertainty Cardiovascular Population Scale	II
			BMQ specific	III
		**Process evaluation**	Experiences of intervention (patient-reported)	IV-V
			Use of intervention (patient-reported, communication lists and platform usage)	IV-V

^a^ Categories according to the ECHO model (for economic, clinical, and humanistic outcomes) [21]

^b^ For study references, see sCase 1–5 in the supplementary material

^c^ Such evaluations include presenting direct costs and sometimes indirect costs

All studies underwent traditional economic evaluations, which have sparked a desire to incorporate the person-centred ethic in cost-effectiveness evaluation. Thus, later study protocols include capability measures (examples from ClinicalTrial.gov: NCT04706195 and NCT04416815). Consistent with other projects from the centre [4], the anticipation and collection of adverse events were not extensive. However, because caring practices can cause adverse events, they should be included in future studies.

The trend toward including process evaluations [22] was only reflected in the later studies (studies IV-V). Transitioning from research conducted in a typical care setting (study I) to supplementary interventions led by the research team (studies II-V), a discernible shift in the study's emphasis can be observed. This shift pertains to how the intervention aligns with usual care (feasibility), shifting toward a focus on the intervention itself (dose) and the individuals being included (reach). Still, intention to treat has remained essential to evaluations to ensure group comparability [23]. Thus, the studies were complementary, exploring the functionality of the intervention within standard care and how it can be delivered.

## Discussion

The studies conducted by staff in usual care and in-house (by research staff), respectively, were mutually supportive, contributing to a deeper comprehension of the efficacy and effectiveness of person-centred care. Initiating, working, and safeguarding a partnership between patient and staff was possible, whether through in-person or remote communication. Evaluations were conducted according to current research standards and built on the experience from the performed studies, thus becoming more comprehensive with time.

This study did not intend to review all publications in this field systematically but focused on projects conducted by the same research group evaluating person-centred care. To ensure the availability of all pertinent information about the projects, researchers who played pivotal roles in each project were included. Nevertheless, it is important to acknowledge that an external researcher may interpret the findings differently. A risk of bias assessment for the studies (we used ROBINS-I [24] for study I and RoB 2.0 [25] for studies II-V) was performed to avoid bias assessments of the projects. We deemed the risk of residual confounding low due to the inclusion of four randomised controlled trials and one non-randomised trial, which had balanced populations and did not exhibit any specific risk of selective recruitment into the study arms. Some concerns were raised because no blinding of a trial arm was possible. The main outcome exhibited a high reporting rate, suggesting minimal bias risk from missing outcome data, though some of the secondary outcomes had a lower reporting rate. Overall, the bias assessment indicated a fairly low level of bias, as expected, and any remaining bias was mainly due to participants not fully adhering to the planned interventions, which should reduce differences in results between study arms rather than result in exaggerated results.

Although a specific research group carried out these projects, it is important to recognise that they were part of a broader research programme initiated by the GPCC during this period [4, 6], based on the original publication operationalising person-centred care [5]. Alongside the work conducted within the research group and collaboration with scientific and ethics reference groups, networking within the research centre added a layer of development through collaborations, seminars, and workshops. Concurrently, the project group played a vital role in the research development of the centre, exemplified by the participation of research group members serving on the steering committee).

### Implications

Given the presence of an ageing population grappling with multiple diagnoses alongside chronic conditions, society must devise and evaluate content that has demonstrated efficacy through the integration of diverse disciplines and methodologies across different contexts. In these complex interventions, collaboration between researchers and patient representatives is crucial. The logic model complements previous models [26, 27] by adding the development from sequential studies. Thus, the extent to which a study can rely on structures and staff from prior projects becomes apparent. This offers insights into the importance of engagement with established frameworks as a novice researcher, highlighting the need for these frameworks to be receptive to novel approaches and adaptable to address societal health care challenges.

In the respective studies, we explored the operationalisation of partnerships between patients and health care professionals in the context of care. Partnership, which encompasses collaboration and mutual respect,

holds utmost significance in person-centred care as it strives to reduce the hierarchical structure within the current care system. For all studies, we hypothesised that person-centred care could improve the quality of care and make it more effective by allowing patients to use their resources and capacities to collaborate with health professionals. The analyses in the present study confirm this assertion, and future studies need to focus more on this important aspect of person-centred care by combining methods from different areas, such as for example health economics, care sciences and medicine.

The assessment of the efficacy of treatment interventions is best achieved through randomised controlled trials (RCTs), which provide the highest evidentiary value. They are widely recognised as the gold standard for evaluation in evidence-based medicine. However, these methods may not always be viable or sufficient for assessing the efficacy of a multifaceted intervention. Quasi-experimental designs (such as study I) may often be the only recourse to evaluate complex interventions. Such designs can provide valuable data on the effectiveness of an intervention to support evidence-based recommendations for everyday clinical practice. The RCT offers a balance between the treatment groups at baseline. However, an intervention such as person-centred care will vary; for various reasons, some participants will not receive person-centred care. In these circumstances, it is necessary to analyse the results through the actual intervention (e.g., per protocol) and complement it with a process evaluation such as that used in studies IV-V. Such an evaluation provides valuable information about critical elements of the complex intervention [28], both in usual care and in understanding alternative delivery methods.

With the accumulation of additional evidence in person-centred care from trials involving diverse patient groups [29] and employing various communication methods, an inevitable progression lies in the expansion towards larger populations and integrating person-centred care into all provider or regional care practices [30]. However, that may imply a return to quasi-experimental designs or even designs lacking a delineated control group alongside routinely gathered data rather than data specifically obtained for the research project.

## Conclusion

To summarise, the development of study design and analyses was ultimately influenced by the cumulative experience gained from previous studies. Such progress is particularly important in the execution of complex interventions. The evaluations were particularly impacted by the emergence of guidelines for cutting-edge research. For instance, there was a noticeable increase in the prevalence of health economics and process evaluations during this timeframe. An approach to consider is the development of large-scale studies on person-centred care involving diverse patient populations, using routinely collected data for assessment.

## Supplementary Information

Below is the link to the electronic supplementary material.

Supplementary file1 (DOCX 80.8 KB)

## Data Availability

No datasets were generated or analysed during the current study.
