# Supplementary material for: Person-Centred Care: State-of-the-Art and Future Perspectives
Source: Curr Heart Fail Rep. 2025 Apr 11;22(1):15. doi: 10.1007/s11897-025-00702-3 (PMC11991961; doi:10.1007/s11897-025-00702-3)
Supplement: Supplementary file 1 — Supplementary file1 (DOCX 80.8 KB) [file 11897_2025_702_MOESM1_ESM.docx]

Person-centred care: State-of-the-art and future perspectives

Hanna Gyllensten^1,2^

Matilda Cederberg^1,2,3^

Sara Alsén^1,2^

Elin Blanck^1,2,4^

Lilas Ali^1,2,5^

Andreas Fors^1,2,6^

Håkan Hedman^2,7^

Laura Pirhonen Nørmark^1,2,8,9^

Karl Swedberg^2,10^

Inger Ekman^1,2,11^

^1^ Institute of Health and Care Sciences, Sahlgrenska Academy, University of Gothenburg, Box 457, SE-405 30 Gothenburg, Sweden.

^2^ Centre for Person-Centred Care (GPCC), Sahlgrenska Academy, University of Gothenburg, Gothenburg, Sweden.

^3^ Department of Psychotic Disorders, Region Västra Götaland, Sahlgrenska University Hospital, Gothenburg, Sweden.

^4^ Faculty of Caring Science, Work Life and Social Welfare, University of Borås, Borås, Sweden.

^5^ Department of Affective Disorders, Region Västra Götaland, Sahlgrenska University Hospital, Gothenburg, Sweden.

^6^ Research, Education, Development and Innovation, Region Västra Götaland, Primary Health Care, Gothenburg, Sweden.

^7^ Swedish Kidney Association, Sweden.

^8^ Department of Economics, Centre for Health Economics (CHEGU), University of Gothenburg, Gothenburg, Sweden.

^9^ Section of Health Services Research, University of Kopenhagen, Kopenhagen, Denmark.

^10^ Department of Clinical and Molecular Medicine, Sahlgrenska Academy, University of Gothenburg, Gothenburg, Sweden.

^11^ Department of Medicine, Geriatrics and Emergency Medicine, Region Västra Götaland, Sahlgrenska University Hospital/Östra, Gothenburg, Sweden.

Content

[**sCase 1**: The PCC-HF study (referred to as Study I) 2](#_Toc150852429)

[**sCase 2**: The PAC study (referred to as Study II) 5](#_Toc150852430)

[**sCase 3**: The C4 study (referred to as Study III) 9](#_Toc150852431)

[**sCase 4**: The PROTECT study (referred to as Study IV) 12](#_Toc150852432)

[**sCase 5**: The PROMISE study (referred to as Study V) 15](#_Toc150852433)

# **sCase 1**: The PCC-HF study (referred to as Study I)

**Aims**

The PCC-HF (person-centred care (PCC) in patients with heart failure (HF)) evaluated the effects of PCC on outcomes in in-hospital patients with HF. The outcomes were the length of hospital stay (LOS), activities of daily living (ADL), health-related quality of life (HRQoL) and hospital readmission within 6 months.

**Methods**

Patients hospitalised with worsening chronic heart failure (CHF) were enrolled if they fulfilled inclusion and exclusion criteria. First, 123 patients were recruited for usual care from five hospital wards while one was a controlled care ward. The experience from this group defined usual care. Based on this experience, a panel of in-house clinicians, researchers and a patient representative developed measures to align usual care with basic PCC principles. These measures were incorporated into a study protocol to guide care procedures at the same five wards. PCC was then implemented at these wards and evaluated in another 125 patients enrolled based on the same exclusion criteria as the usual care group. LOS and 6-month readmission were extracted from patient records. ADL was evaluated at baseline and discharge and HRQoL at baseline and 3 months after admission.

**Results**

In the analysis of all patients LOS was reduced by 1 day (p=0.16) while retaining ADL (p=0.07). When PCC was fully implemented (per protocol analysis), LOS was reduced by 2.5 days (p=0.01) and the ADL level was better preserved (p=0.04). HRQoL and time-to-first readmission did not differ.

**Conclusions**

Our findings suggest that a fully implemented PCC approach shortens hospital stay and maintains functional performance in patients hospitalised for worsening CHF without increasing the risk of readmission or jeopardising patients’ HRQoL.^1^

**Additional findings**

One paper showed that PCC can result in lower levels of uncertainty in illness.^2^ The discharge process is improved and more efficient when PCC is used, partly because patients are considered competent enough to be involved in planning their care.^3^ A cost-utility analysis was performed where the costs and effects of the intervention were compared with usual care, in which the intervention was cheaper than usual care.^4^ One year after the intervention, an interview study explored patients’ partnership experiences in the hospital wards. The paper showed that the patients were not interested in participating and being active in their care. This unwillingness could be partly explained by the fact that it was difficult for the staff to invite them and increase their confidence in their ability.^5^ The organisational culture was studied, and the results indicate that a culture in which stability is valued benefits PCC implementation.^6^ The impact of organisational culture on the patient’s quality of life was also examined.^7^ The implementation process was studied, with results indicating that the implementation was incomplete and there was a risk of slipping into old routines.^8^

**References**

1. Ekman I, Wolf A, Olsson LE, et al. Effects of person-centred care in patients with chronic heart failure: the PCC-HF study. *Eur Heart J*. May 2012;33(9):1112-9. doi:10.1093/eurheartj/ehr306

2. Dudas K, Olsson LE, Wolf A, et al. Uncertainty in illness among patients with chronic heart failure is less in person-centred care than in usual care. *Eur J Cardiovasc Nurs*. Dec 2013;12(6):521-8. doi:10.1177/1474515112472270

3. Ulin K, Olsson LE, Wolf A, Ekman I. Person-centred care - An approach that improves the discharge process. *Eur J Cardiovasc Nurs*. Apr 2016;15(3):e19-26. doi:10.1177/1474515115569945

4. Hansson E, Ekman I, Swedberg K, et al. Person-centred care for patients with chronic heart failure - a cost-utility analysis. *Eur J Cardiovasc Nurs*. Jun 2016;15(4):276-84. doi:10.1177/1474515114567035

5. Alharbi TS, Carlström E, Ekman I, Jarneborn A, Olsson LE. Experiences of person-centred care - patients' perceptions: qualitative study. *BMC Nurs*. Oct 2014;13:28. doi:10.1186/1472-6955-13-28

6. Alharbi TS, Ekman I, Olsson LE, Dudas K, Carlstrom E. Organizational culture and the implementation of person centered care: results from a change process in Swedish hospital care. *Health Policy*. Dec 2012;108(2-3):294-301. doi:10.1016/j.healthpol.2012.09.003

7. Alharbi TS, Olsson LE, Ekman I, Carlström E. The impact of organizational culture on the outcome of hospital care: after the implementation of person-centred care. *Scand J Public Health*. Feb 2014;42(1):104-10. doi:10.1177/1403494813500593

8. Alharbi TS, Carlström E, Ekman I, Olsson L-E. Implementation of person-centred care: management perspective. *J Hosp Admin*. May 2014;3(3):107. doi: 10.5430/jha.v3n3p107

# **sCase 2**: The PAC study (referred to as Study II)

**Aims**

The PAC (person-centred care [PCC] in patients with acute coronary syndrome [ACS]) study evaluated the effects of a person-centred intervention throughout the care chain after an ACS event.

**Methods**

A randomised controlled trial (RCT) was conducted between 2011 and 2014. The trial enrolled 199 patients (105 control patients, 94 intervention patients) with ACS admitted to either of two hospital sites within the Sahlgrenska University Hospital catchment area. The intervention group received PCC in addition to usual care, in which care was co-created between patients and health care professionals across three health care levels (hospital, outpatient, and primary care) and documented in a health plan, integrating patient’s resources and needs with medical expertise to achieve agreed goals. Patients were offered an eHealth diary and a symptom-tracking tool as an optional supplement to self-rate their symptoms. The primary outcome was a composite score of changes comprising general self-efficacy, return to work or previous activity level and re-hospitalisation or death at the 6-month follow-up. In addition, per-protocol, secondary outcome, and sub-group analysis were performed. Data from the follow-ups at 4 and 8 weeks and 6 and 24 months were analysed.

**Results**

At the 6-month follow-up, more participants in the PCC group improved in the composite score compared to the usual care group (p=0.015). The effect was driven by more patients in the PCC group improving their general self-efficacy with ≥5 units (p=0.026). No significant between-group differences were observed on re-hospitalisation or death, return to work, or prior activity level.

**Conclusions**

In this RCT our findings suggest that a PCC approach in which patients and health care professionals collaborate as partners improves patients’ levels of self-efficacy without causing worsening clinical events.^1^

**Additional findings**

The effect in improved general self-efficacy was sustained after controlling for socio-economic and disease-related variables^2^ and at the 2-year follow-up.^3^ The patients (40%) who used the eHealth diary and symptom-tracking tool combined with the PCC intervention reported an even higher improvement in the composite score compared to the control group.^4^ At the 8-week follow-up, the PCC group reported improved scores in the control symptoms dimension in the cardiac self-efficacy scale^6^ (psychometrically evaluated based on PAC data^5^). In contrast, the control group reported worsening scores.^6^ The person-centred intervention improved patients´ experiences of information, access to documentation and involvement of family and friends. This effect was most prominent in patients without post-secondary education.^7^ Sub-group analysis also showed that in the group of patients without post-secondary education, a higher number of patients who received the PCC intervention improved according to the composite score, indicating that the intervention was especially beneficial for patients without post-secondary education.^8^ Findings from an interview study revealed that patients affected by ACS construct personal models to explain their disease, emphasising the importance of co-creating health plans together with health care professionals based on these models.^9^ A descriptive study of the content of the health plan findings showed that the PCC intervention enabled patients to set realistic goals and identify their resources and family and social support systems.^10^ The PCC intervention was more cost-effective than usual care in patients <65 years,^11^ and also in a 2- and 5-year perspective.^12^ Ongoing/future work explores how the intervention potentially affects drug use.

**References**

1. Fors A, Ekman I, Taft C, et al. Person-centred care after acute coronary syndrome, from hospital to primary care - A randomised controlled trial. *Int J Cardiol*. May 2015;187:693-9. doi:10.1016/j.ijcard.2015.03.336

2. Pirhonen L, Olofsson EH, Fors A, Ekman I, Bolin K. Effects of person-centred care on health outcomes-A randomized controlled trial in patients with acute coronary syndrome. *Health Policy*. Feb 2017;121(2):169-179. doi:10.1016/j.healthpol.2016.12.003

3. Fors A, Swedberg K, Ulin K, Wolf A, Ekman I. Effects of person-centred care after an event of acute coronary syndrome: Two-year follow-up of a randomised controlled trial. *Int J Cardiol*. Dec 2017;249:42-47. doi:10.1016/j.ijcard.2017.08.069

4. Wolf A, Fors A, Ulin K, Thorn J, Swedberg K, Ekman I. An eHealth Diary and Symptom-Tracking Tool Combined With Person-Centered Care for Improving Self-Efficacy After a Diagnosis of Acute Coronary Syndrome: A Substudy of a Randomized Controlled Trial. *J Med Internet Res*. Feb 2016;18(2):e40. doi:10.2196/jmir.4890

5. Fors A, Ulin K, Cliffordson C, Ekman I, Brink E. The Cardiac Self-Efficacy Scale, a useful tool with potential to evaluate person-centred care. *Eur J Cardiovasc Nurs*. Dec 2015;14(6):536-43. doi:10.1177/1474515114548622

6. Fors A, Taft C, Ulin K, Ekman I. Person-centred care improves self-efficacy to control symptoms after acute coronary syndrome: a randomized controlled trial. *Eur J Cardiovasc Nurs*. Apr 2016;15(2):186-94. doi:10.1177/1474515115623437

7. Wolf A, Vella R, Fors A. The impact of person-centred care on patients' care experiences in relation to educational level after acute coronary syndrome: secondary outcome analysis of a randomised controlled trial. *Eur J Cardiovasc Nurs*. Apr 2019;18(4):299-308. doi:10.1177/1474515118821242

8. Fors A, Gyllensten H, Swedberg K, Ekman I. Effectiveness of person-centred care after acute coronary syndrome in relation to educational level: Subgroup analysis of a two-armed randomised controlled trial. *Int J Cardiol*. Oct 2016;221:957-62. doi:10.1016/j.ijcard.2016.07.060

9. Fors A, Dudas K, Ekman I. Life is lived forwards and understood backwards--experiences of being affected by acute coronary syndrome: a narrative analysis. *Int J Nurs Stud*. Mar 2014;51(3):430-7. doi:10.1016/j.ijnurstu.2013.06.012

10. Jansson I, Fors A, Ekman I, Ulin K. Documentation of person-centred health plans for patients with acute coronary syndrome. *Eur J Cardiovasc Nurs*. Feb 2018;17(2):114-122. doi:10.1177/1474515117718415

11. Pirhonen L, Bolin K, Olofsson EH, et al. Person-Centred Care in Patients with Acute Coronary Syndrome: Cost-Effectiveness Analysis Alongside a Randomised Controlled Trial. *Pharmacoecon Open*. Dec 2019;3(4):495-504. doi:10.1007/s41669-019-0126-3

12. Pirhonen L, Gyllensten H, Fors A, Bolin K. Modelling the cost-effectiveness of person-centred care for patients with acute coronary syndrome. *Eur J Health Econ*. Dec 2020;21(9):1317-1327. doi:10.1007/s10198-020-01230-8

# **sCase 3**: The C4 study (referred to as Study III)

**Aims**

The C4 (Care4Ourselves) study evaluated the effects of person-centred support at a distance via telephone support for patients with chronic obstructive pulmonary disease (COPD) and/or chronic heart failure (CHF).

**Methods**

A total of 221 patients ≥50 years old with COPD and/or CHF were randomised to usual care or person-centred care (PCC) in the form of structured telephone support in addition to routine care and followed for 6 months. In the intervention group, a registered nurse telephoned patients to co-create a health plan reflecting both perspectives. The health plan was further discussed and evaluated during additional telephone follow-ups. At the 6-month follow-up, a composite score of changes that included changes in general self-efficacy ≥5 units, re-hospitalisation. and death was used as the primary outcome measure.

**Results**At the 6-month follow-up, no difference in the composite score was found between the two study groups (p=0.102). Significantly more patients in the control group decreased ≥5 units in general self-efficacy (p=0.011). There were no significant differences between groups in re-hospitalisation or death. In the per-protocol analysis of the composite score more patients in the control group deteriorated compared to the intervention group (p=0.039).

**Conclusions**

Person-centred support at a distance through telephone support mitigates worsened self-efficacy without increasing the risk of clinical events in patients with CHF and/or COPD. This finding highlights the possibility of establishing a patient-health care professional partnership that is not dependent on face-to-face encounters, even in vulnerable patient groups.^1^

**Additional findings**

An interview study showed that people with COPD found their strength by sharing experiences with others having COPD and with health care professionals.^2^ Informal carers to relatives with COPD and/or CHF want to be involved as carers in partnership.^3^ The results of two sub-group analyses showed that self-reported fatigue was reduced in patients with CHF in the intervention group,^4^ and that confidence to manage the illness improved in patients with COPD in the intervention group.^5^ Exploratory analysis showed that an increased or sustained self-efficacy was associated with lower directs costs in patients with CHF and/or COPD.^6^ The registered nurses who conducted the person-centred telephone support needed to remould their professional role to involve patients as partners in their care.^7^ PCC improved health-related quality of life and resulted in lower costs compared to usual care.^8^ In a descriptive study of the content of the health plans the results indicated that emphasising attentive listening, which is provided by person-centred telephone care, facilitates the patient’s goals, interventions, and resources. These elements can be used to customise support and involve patients as active partners in their care.^9^ Ongoing/future work explores the building of a partnership in an intervention like this.

**References**1. Fors A, Blanck E, Ali L, et al. Effects of a person-centred telephone-support in patients with chronic obstructive pulmonary disease and/or chronic heart failure - A randomized controlled trial. *PLoS One*. Aug 2018;13(8):e0203031. doi:10.1371/journal.pone.0203031
2. Ali L, Fors A, Ekman I. Need of support in people with chronic obstructive pulmonary disease. *J Clin Nurs*. Mar 2018;27(5-6):e1089-e1096. doi:10.1111/jocn.14170
3. Blanck E, Fors A, Ali L, Brännström M, Ekman I. Informal carers in Sweden - striving for partnership. *Int J Qual Stud Health Well-being*. Dec 2021;16(1):1994804. doi:10.1080/17482631.2021.1994804
4. Wallström S, Ali L, Ekman I, Swedberg K, Fors A. Effects of a person-centred telephone support on fatigue in people with chronic heart failure: Subgroup analysis of a randomised controlled trial. *Eur J Cardiovasc Nurs*. Jun 2020;19(5):393-400. doi:10.1177/1474515119891599
5. Ali L, Wallström S, Ekman I, Swedberg K, Fors A. Effects of person-centred care via telephone on self-efficacy in patients with chronic obstructive pulmonary disease: Subgroup analysis of a randomized controlled trial. *Nurs Open*. Mar 2021;8(2):927-935. doi:10.1002/nop2.701
6. Blanck E, Pirhonen Nørmark L, Fors A, Ekman I, Ali L, Swedberg K, Gyllensten H. Self-efficacy and healthcare costs in patients with chronic heart failure or chronic obstructive pulmonary disease. *ESC Heart Fail*. Feb 2024;11(1):219-228. doi: 10.1002/ehf2.14574. 
7. Boström E, Ali L, Fors A, Ekman I, Andersson AE. Registered nurses' experiences of communication with patients when practising person-centred care over the phone: a qualitative interview study. *BMC Nurs*. Jun 2020;19:54. doi:10.1186/s12912-020-00448-4
8. Pirhonen L, Gyllensten H, Olofsson EH, et al. The cost-effectiveness of person-centred care provided to patients with chronic heart failure and/or chronic obstructive pulmonary disease. *Health Policy Open*. Dec 2020;1:100005. doi:10.1016/j.hpopen.2020.100005
9. Ulin K, Fors A, Ali L, Ekman I, Jansson I. Flip focus and emphasise patient resources in person-centred care over the telephone-A retrospective descriptive study. *Scand J Caring Sci*. Sep 2023;37(3):797-804. doi:10.1111/scs.13164.

# **sCase 4**: The PROTECT study (referred to as Study IV)

**Aims**

The PROTECT (Person-centred care [PCC] at a distance) study investigated the effects of PCC through a combined digital platform and telephone support for people with chronic obstructive pulmonary disease (COPD) and/or chronic heart failure (CHF).

**Methods**

In a randomised controlled trial 222 patients with a diagnosis of COPD, CHF, or both were recruited from nine primary care centres between 2018 and 2020. Participants were randomised into usual care (n=112) or PCC combined with usual care (n=110). The telephone calls were based on the patient’s narrative and formed the basis for co-creating a personal health plan, which was uploaded to the digital platform. Patients could rate and monitor their symptoms and progression on the platform, read health plans, chat with health care professionals, and acquire information about their condition. The primary endpoint was a composite score of changes in general self-efficacy and re-hospitalisation or death at a 6-month follow-up. Intention-to-treat and per-protocol analysis (PP) (those participants who used the intervention) were conducted at 3- and 6-month follow-ups, along with a process evaluation.

**Results**

No significant differences were found between the groups in the composite score at the 3- and 6-month follow-ups. However, a significant difference between the groups favouring the intervention was seen in the PP analysis of the 3-month follow-up (p=0.047). The effect was driven by a change in general self-efficacy from baseline, but the difference was not sustained at the 6-month follow-up (p=0.240).

**Conclusions**

By structured telephone support in combination with a digital platform, PCC seems to support people with COPD and/or CHF to increase their short-term self-efficacy beliefs.^1^

**Additional findings**

The protocol for the study is published.^2^ There was also a process evaluation in this project, which examined the patients’ experiences of participating in the intervention. The remote intervention was a feasible approach to support patients’ self-management and involvement in preventive care.^3^ Moreover, the intervention was considered meaningful, especially by patients with COPD. Telephone support was used to a greater extent and was reported as more meaningful than the digital platform.^4^ An interview study revealed that patients experience medicines in five key ways, ranging from essential to problematic, and these findings support mutually agreed prescribing through co-creative dialogue between patients and healthcare professionals.^5^ Ongoing/future work explores the health plan’s content, how the intervention potentially affects drug use and other secondary outcomes for up to 2 years.

**References**

1. Ali L, Wallström S, Fors A, et al. Effects of Person-Centered Care Using a Digital Platform and Structured Telephone Support for People With Chronic Obstructive Pulmonary Disease and Chronic Heart Failure: Randomized Controlled Trial. *J Med Internet Res*. Dec 2021;23(12):e26794. doi:10.2196/26794

2. Ali L, Wallström S, Barenfeld E, et al. Person-centred care by a combined digital platform and structured telephone support for people with chronic obstructive pulmonary disease and/or chronic heart failure: study protocol for the PROTECT randomised controlled trial. *BMJ Open*. Jul 2020;10(7):e036356. doi:10.1136/bmjopen-2019-036356

3. Barenfeld E, Ali L, Wallström S, Fors A, Ekman I. Becoming more of an insider: A grounded theory study on patients' experience of a person-centred e-health intervention. *PLoS One*. Nov 2020;15(11):e0241801. doi:10.1371/journal.pone.0241801

4. Barenfeld E, Fuller JM, Wallström S, Fors A, Ali L, Ekman I. Meaningful use of a digital platform and structured telephone support to facilitate remote person-centred care - a mixed-method study on patient perspectives. *BMC Health Serv Res*. Apr 2022;22(1):442. doi:10.1186/s12913-022-07831-8

5. Fuller JM, Barenfeld E, Ekman I. Why do patients struggle with their medicines? – A phenomenological hermeneutical study of how patients experience medicines in their everyday lives. *PLoS One*. Aug 2021;16(8):e0255478. doi: 10.1371/journal.pone.0255478. eCollection 2021.

# **sCase 5**: The PROMISE study (referred to as Study V)

**Aims**

The PROMISE (Person-centred eHealth for treatment and rehabilitation of common mental disorders) study studied the effects of person-centred eHealth support (telephone calls and eHealth platform) for patients on sick leave for common mental disorders (CMDs).

**Methods**

A randomised controlled study managed remotely included 209 patients randomly assigned to either a control (n=107) or an intervention group (n=102). The participants in the control group received care as usual and the intervention group received care as usual, plus person-centred care through person-centred eHealth support. The purpose of the intervention was to enable partnerships between patients, significant others, and health care professionals in a remote context. The intervention structure was identical to that in the PROTECT project, i.e., patients in the intervention group received phone support (scheduled or on-demand during office hours) and access to a web-based portal as an addition to usual care to alleviate self-management. The content was adapted to accommodate the conditions included in the PROMISE project.

The primary outcome was a composite score of changes in general self-efficacy and level of sick leave at a 6-month follow-up. Data were analysed at the 3- and 6-month follow-ups also including per-protocol analyses (participants who had at least one phone call and used the web-based portal at least once)

**Results**

At the 3-month follow-up, there was a significant improvement in the composite score in the intervention group compared with the control group (p=0.04), but the between-group difference was no longer significant at the 6-month follow-up (p=0.22). In the per-protocol analysis the difference between the groups was significant at 3 months (p=0.02) but not at 6 months (p=0.06). The effect was driven by a significant improvement in general self-efficacy scores. However, the level of sick leave did not differ between the groups in any analysis or the two follow-ups.

**Conclusions**

The person-centred eHealth intervention increased general self-efficacy, most strongly after 3 months but did not affect the level of sick leave.^1^

**Additional findings**

A published study protocol has further described the intervention and overall project design.^2^ Results based on secondary outcome analysis showed that person-centred eHealth support reduced symptoms of burnout.^3^ In two interview studies patients’ experiences of being affected by stress-related exhaustion and support in this situation have been explored. The findings show that people with stress-related exhaustion experience existential challenges and need allies in their struggle for acknowledgement as persons.^4^ Persons affected by stress-related exhaustion required a caring dialogue to deal with feelings of shame, guilt, and meaninglessness.^5^ A conversation analysis exploring interactive practices in phone conversations revealed that practising PCC and employing narrative elicitation requires sensitivity, communicative skills, and adaptability to accommodate the different narrative styles that patients and interactions may present.^6^ A process evaluation using mixed methods showed that most of the participants found the intervention to be either partially or entirely meaningful. The phone calls with healthcare professionals within the intervention were identified as the most meaningful activity.^7^ Ongoing/future work explores efficacy, cost-effectiveness, and other secondary outcomes for up to 2 years.

**References**

1. Cederberg M, Alsén S, Ali L, et

al. Effects of a Person-Centered eHealth Intervention for Patients on Sick Leave Due to Common Mental Disorders (PROMISE Study): Open Randomized Controlled Trial. *JMIR Ment Health*. Mar 2022;9(3):e30966. doi:10.2196/30966

2. Cederberg M, Ali L, Ekman I, et al. Person-centred eHealth intervention for patients on sick leave due to common mental disorders: study protocol of a randomised controlled trial and process evaluation (PROMISE). *BMJ Open*. Sep 2020;10(9):e037515.

doi:10.1136/bmjopen-2020-037515

# 3. Alsén S, Hadžibajramović E, Jonsdottir IH, Ali L, Fors A. Effectiveness of a person-centred eHealth intervention in reducing symptoms of burnout in patients with common mental disorders - secondary outcome analysis of a randomized controlled trial. *BMC Prim Care*. Oct 2023;24(1):210. doi: 10.1186/s12875-023-02172-9.

4. Alsén S, Ali L, Ekman I, Fors A. Having allies-Experiences of support in people with stress-related exhaustion: A qualitative study. *PLoS One*. Nov 2022;17(11):e0277264. doi:10.1371/journal.pone.0277264

5. Alsén S, Ali L, Ekman I, Fors A. Facing a blind alley - Experiences of stress-related exhaustion: a qualitative study. *BMJ Open*. Sep 2020;10(9):e038230. doi:10.1136/bmjopen-2020-038230

6. Cederberg M, Fors A, Ali L, Goulding A, Mäkitalo Å. The interactive work of narrative elicitation in person-centred care: Analysis of phone conversations between health care professionals and patients with common mental disorders. *Health Expect*. Jun 2022;25(3):971-983. doi:10.1111/hex.13440
7. Cederberg M, Barenfeld E, Ali L, Ekman I, Goulding A, Fors A. A lowered threshold to partnerships: a mixed methods process evaluation of participants’ experiences of a person-centred eHealth intervention. *BMC Health Serv Res*. Nov 2023;23(1):1193. doi: 10.1186/s12913-023-10190-7.
